# Changes in PEDF, MMP-2, and TGF-β2 levels in the aqueous humor of cataract patients and their correlation with disease severity

**DOI:** 10.1016/j.clinsp.2024.100402

**Published:** 2024-10-16

**Authors:** Yong Feng Lin, Jin Xia Xie, Xiao Luan Chen

**Affiliations:** a Department of Ophthalmology, Longyan People's Hospital, Longyan City, Fujian Province, China; b Department of General Surgery, Longyan Hospital of Traditional Chinese Medicine affiliated to Xiamen University, Longyan City, Fujian Province, China

**Keywords:** Cataract, Aqueous Humor, Pigment Epithelial-Derived Factor, Matrix Metalloproteinase-2, Transforming Growth Factor-β2, LOCS III Classification

## Abstract

**Objective:**

To explore the changes of Pigment Epithelium-Derived Factor (PEDF), Matrix Metalloproteinase-2 (MMP-2), and Transforming Growth Factor-β2 (TGF-β2) levels in the aqueous humor of cataract patients and their correlation with disease severity.

**Methods:**

93 cataract patients and 56 healthy subjects were study objects. PEDF, MMP-2, and TGF-β2 levels of aqueous humor were compared, and the correlation between each index and Lens Opacity Classification System (LOCS) III classification was analyzed. ROC curve was used to analyze the evaluation value of the combined detection of each index on cataract development, and logistic regression to analyze the influence of the changes of each index on cataract development.

**Results:**

PEDF levels were lower and MMP-2 and TGF-β2 levels were higher in the aqueous humor of cataract patients than in healthy subjects. PEDF levels in the aqueous humor were negatively correlated with LOCS III classification, while MMP-2 and TGF-β2 levels were positively correlated with LOCS III classification. The AUC value of combined detection was higher than that of PEDF, MMP-2, and TGF-β2 in the aqueous humor alone. MMP-2 ≥ 15.13 pg/mL, TGF-β2 ≥ 385.91 pg/mL and PEDF < 198.85 ng/mL were risk factors for cataract development.

**Conclusion:**

The changes in PEDF, MMP-2, and TGF-β2 levels in the aqueous humor of cataract patients are related to LOCS III classification. The combined detection is valuable in evaluating cataract development.

## Highlights

• PEDF levels were lower and MMP-2 and TGF-β2 levels were higher in cataracts.
• The combined detection was valuable in evaluating cataract development.
• MMP-2, TGF-β2, and PEDF were risk factors for cataract development.

## Introduction

Cataract refers to a visual disorder caused by factors such as aging, heredity, local nutritional disorders, and immune and metabolic abnormalities, which can cause lens metabolism disorders and lead to lens protein degeneration and turbidity. The onset of this disease is slow, and it is more common in elderly patients. The main symptom of patients is painless progressive vision loss.^1^ Previous reports have indicated the significance of inflammatory cytokines in cataracts.^2^^,^^3^ Matrix metalloproteinases and inhibitors of metalloproteinases have important roles in the development and progression of ocular diseases and can be used as pharmacological targets for the treatment of these diseases.^4^ Matrix Metalloproteinase-2 (MMP-2) is an extracellular matrix protease in the metalloproteinase family, which is mainly involved in the hydrolysis of collagen and other extracellular matrix proteins.^5^ Clinical data have shown that MMP-2 may be involved in remodeling the extracellular matrix and regulating the function of lens epithelial cells.^6^ Transforming Growth Factor-β2 (TGF-β2) is also a common inflammatory factor that can regulate the inflammatory process in the body.^7^ It has been shown that TGF-β2 regulates both physiologic and pathological conditions of the lens,^8^ which is involved in cataract development.^9^ In addition, it has been shown that TGF-β2 levels in the atrial fluid are elevated in patients with primary open-angle glaucoma.^10^ Pigment Epithelial-Derived Factor (PEDF) is an angiogenesis inhibitor, which can inhibit the formation of various neovascularization cells.^11^ Downregulation of PEDF expression can lead to lens aging, and the downregulation degree is further aggravated with the increase of lens opacity.^12^ Downregulation of the PEDF gene in human lens epithelium cells changed the expression of proteins vimentin and alphaB-crystallin.^13^ Furthermore, PEDF expression decreases with age.^14^ Based on this, this study explores the changes of PEDF, MMP-2, and TGF-β2 levels in the aqueous humor of cataract patients and the correlation scores with cataract progression, to provide a reference for the clinical evaluation of cataracts.

## Data and methods

### Clinical data

A total of 93 cataract patients and 56 healthy subjects treated in Longyan People's Hospital from January 2020 to December 2022 were selected as the study objects. Inclusion criteria for cataract group: 1) Cataract history greater than 1 year; 2) Intact posterior capsule lentis; 3) Complete clinical data. Exclusion criteria for cataract group: 1) Patients with proliferative diabetic retinal vascular disease; 2) Patients with severe xerophthalmia; 3) Patients with ocular trauma, surgery, and fundus laser history; 4) Patients with infectious diseases; 5) Combined with optic neuritis, uveitis, high myopia, glaucoma. The control group was selected from apparently healthy people who were patients' companions who came to the same ophthalmological center. They did not any obvious symptoms or diagnoses of diseases at the study time as reported by themselves. Cataract diagnosis in the cataract group has been done based on the opinion of the expert ophthalmologist according to the slit lamp procedure.^15^

This study was an Observational clinical study, following Strengthening the Reporting of Observational Studies in Epidemiology (STROBE) guidelines. The study was approved by the ethics committee of Longyan People's Hospital (n° 201910FJ202) and informed consent from each patient was obtained beforehand. This study adhered to the tenets of the Declaration of Helsinki.

### Methods

Under sterile conditions, 0.1 mL aqueous humor was extracted by anterior chamber puncture using a syringe with a 25-gauge needle 1 mm inside the corneal limbo, and attention was paid to avoiding damage to intraocular tissue during the puncture. PEDF, MMP-2, and TGF-β2 in the aqueous humor were detected by ELISA kits (Beijing Baiaolaibo Technology Co., Ltd.).

### Observation indicators

PEDF, MMP-2, and TGF-β2 levels of aqueous humor were assessed. Cataract type and severity were graded using the LOCS III,^16^ Specifically, patients were categorized as Nuclear Opalescence (NO) II‒V. The correlation between each index and cataract severity and the evaluation value of cataract severity were analyzed.

### Statistical analysis

SPSS 22.0 software was utilized to process the data, the statistical data were expressed as percentages, and the difference between groups was compared by χ^2^ test. Measurement data were expressed by ($\bar{x}$ ± s) after the normal test, and the differences between groups were compared by *t*-test. Spearman test was utilized to analyze the correlation between PEDF, MMP-2, and TGF-β2 levels in the aqueous humor and LOCS III classification, ROC curve to analyze the evaluation value of PEDF, MMP-2, and TGF-β2 levels on cataract development, and logistic regression to analyze the effects of PEDF, MMP-2, and TGF-β2 levels on cataract development; p < 0.05 meant a significant difference.

## Results

### Clinical data

Table 1 hound no significant difference in clinical data between cataract patients and healthy subjects (p > 0.05).

**Table 1:** Comparison of clinical data between cataract group and control group.

Items		Cataract group (n = 93)	Control group (n = 56)	χ2/t	p
Gender	Male	50 (53.8 %)	31 (55.4 %)	0.036	0.850
	Female	43 (46.2 %)	25 (44.6 %)		
Age (years)		58.67 ± 10.59	59.01±11.35	0.185	0.854
Myopic ophthalmopathy		20 (21.5 %)	11 (19.6 %)	0.074	0.786
Hyperopia		25 (26.9 %)	12 (21.4 %)	0.557	0.456
Combined underlying disease	Hypertension	21 (22.6 %)	9 (16.1 %)	0.921	0.337
	Coronary heart disease	15 (16.1 %)	8 (14.3 %)	0.091	0.763
Cataract duration (years)		2.15 ± 0.43	/		
Best corrected visual acuity		0.40 ± 0.18	/		
LOCS III	NO II	38 (40.9 %)	/		
	NO III	24 (25.8 %)	/		
	NO IV	16 (17.2 %)	/		
	NO V	15 (16.1 %)	/		
Affected eyes	Left	41 (44.1 %)	/		
	Right	49 (52.7 %)	/		
	Left and right	3 (3.2 %)	/		

### Evaluation of PEDF, MMP-2, and TGF-β2 levels in the aqueous humor

**Fig. 1:**
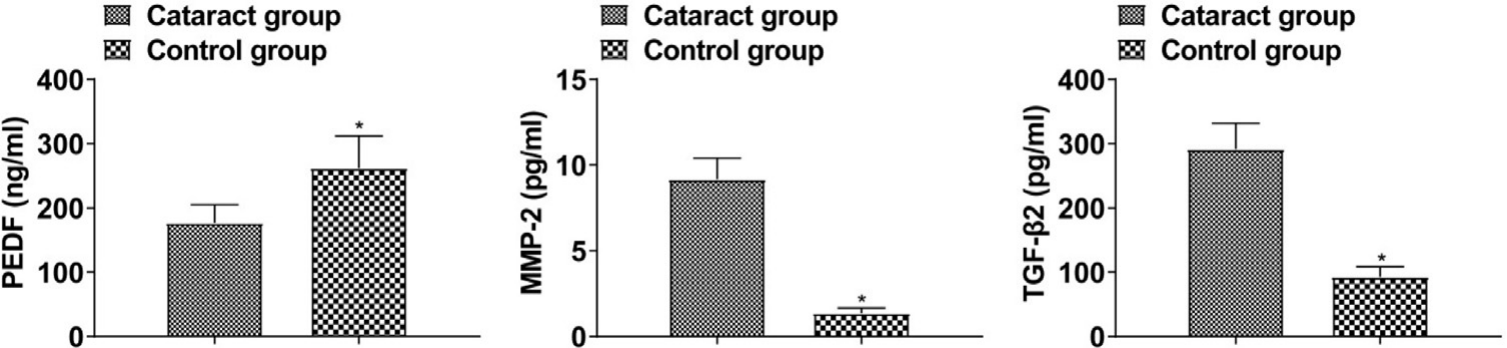
PEDF, MMP-2, and TGF-β2 levels in cataract patients and healthy subjects (*p < 0.05 vs. control group).

PEDF was lower and MMP-2 and TGF-β2 were higher in the aqueous humor of cataract patients than that of healthy subjects (p < 0.05, Fig. 1).

### PEDF, MMP-2, and TGF-β2 levels in patients with different LOCS III classification

**Fig. 2:**
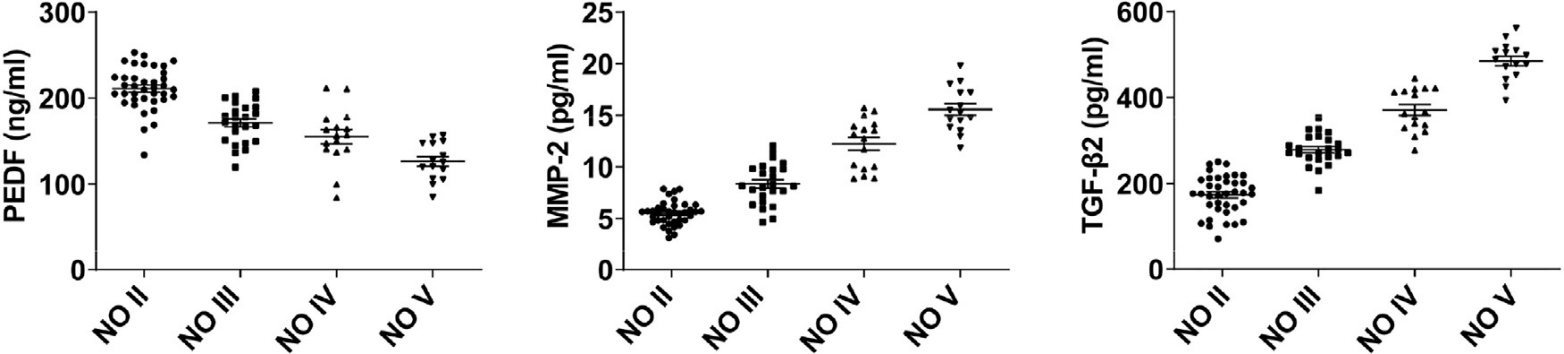
PEDF, MMP-2, and TGF-β2 levels in the aqueous humor of patients with different LOCS III classification.

PEDF level in the aqueous humor in the NO II, NO III, NO IV, and NO V showed a downward trend, and MMP-2 and TGF-β2 showed an upward trend (p < 0.05, Fig. 2).

### Correlation analysis

PEDF level was negatively correlated while MMP-2 and TGF-β2 levels were positively correlated with LOCS III classification (p < 0.05, Table 2), suggesting that PEDF, MMP-2 and TGF-β2 levels in the aqueous humor may be associated with disease severity.

**Table 2:** Spearman analysis of the correlation between PEDF, MMP-2 and TGF-β2 levels in aqueous humor and LOCS III classification.

Indicators	LOCS III	
	*r*	p
PEDF	-0.618	< 0.001
MMP-2	0.632	< 0.001
TGF-β2	0.659	< 0.001

### Evaluation value of PEDF, MMP-2, and TGF-β2 levels in the aqueous humor

**Fig. 3:**
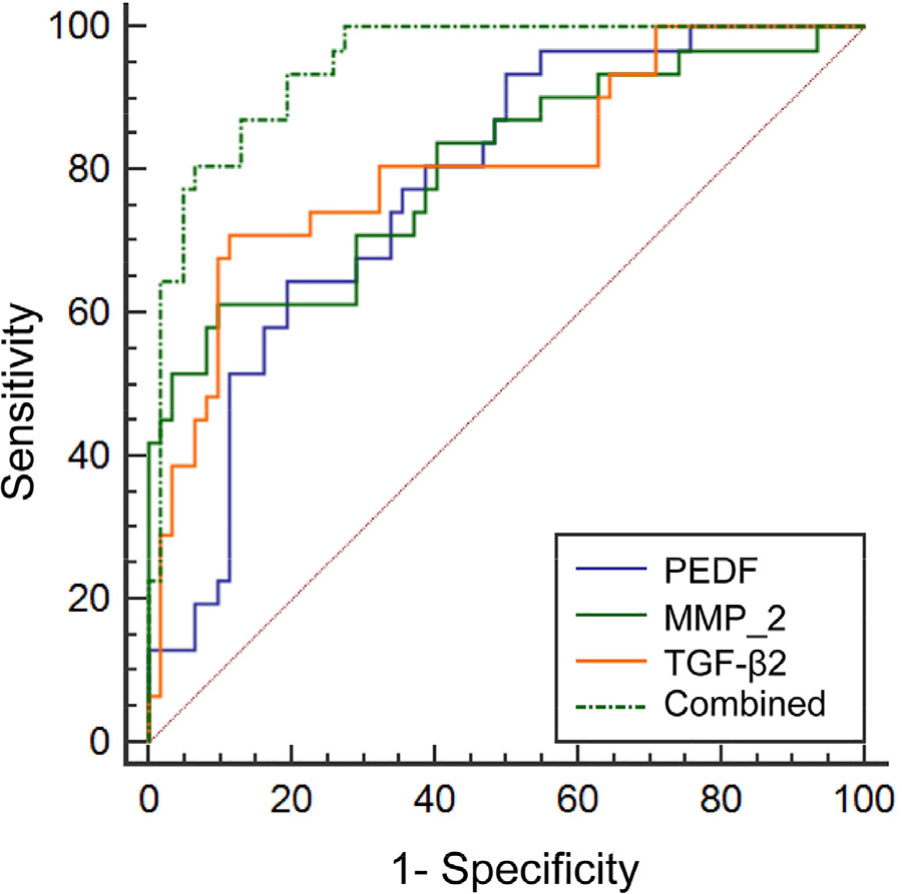
ROC curve of PEDF, MMP-2, and TGF-β2 levels in the aqueous humor.

The AUC value of the combined detection in evaluating cataract development was greater than that of the single detection of PEDF, MMP-2, and TGF-β2 levels in the aqueous humor (p < 0.05, Table 3, Fig. 3).

**Table 3:** Evaluation value of PEDF, MMP-2 and TGF-β2 levels in aqueous humor.

Indicators	Cut-off value	AUC	SE	95 %CI
PEDF	198.85 ng/mL	0.777^a^	0.049	0.679∼0.857
MMP-2	15.13 pg/mL	0.803^a^	0.052	0.707∼0.878
TGF-β2	385.91 pg/mL	0.809^a^	0.051	0.714∼0.883
Combined		0.947	0.021	0.880∼0.983

Note: Compared with combined,

a p < 0.05.

### Logistic regression analysis of PEDF, MMP-2, and TGF-β2 levels in the aqueous humor

MMP-2 ≥ 15.13 pg/mL, TGF-β2 ≥ 385.91 pg/mL and PEDF < 198.85 ng/mL were the risk factors affecting cataract development (p < 0.05, Table 4), indicating that elevated levels of MMP-2 and TGF-β2 and reduced levels of PEDF may be involved in cataract development.

**Table 4:** Logistic regression analysis of PEDF, MMP-2 and TGF-β2 levels in aqueous humor.

Indicators	β	SE	wald χ^2^	OR	95 %CI	p
PEDF	-0.049	0.015	10.671	0.952	0.925∼0.981	0.001
MMP-2	0.647	0.234	7.645	1.91	1.207∼3.021	0.006
TGF-β2	0.039	0.01	15.21	1.04	1.020∼1.060	0.000
Constant	-1.303	0.493	6.985	0.272	0.103∼0.714	0.009

Assignment: PEDF (≥ 198.85 ng/mL was 1, < 198.85 ng/mL was 0); MMP-2 (≥ 15.13 pg/mL was 1, < 15.13 pg/mL was 0); TGF-β2 (≥ 385.91 pg/mL was 1, < 385.91 pg/mL was 0).

## Discussion

Cataracts are visual disorders caused by lens opacity. The lens is an optical component in the human eyeball and is normally transparent.^17^^,^^18^ Due to various reasons, the protein of the lens is denatured and cloudy, resulting in varying degrees of vision loss in patients. Relevant data show that there are many factors inducing cataracts in the elderly.^19^ Some studies have shown that inflammation is closely related to cataract disease.^20^^,^^21^ MMPs are inflammatory factors in the body and a group of endogenous proteolytic enzymes that can degrade extracellular matrix components. The dynamic balance between the production and degradation of matrix regulates wound repair and tissue reconstruction, and its role in tissue fibrosis has attracted much attention in recent years.^22^^,^^23^ MMP-2 is a kind of matrix metalloproteolytic enzyme mainly based on type IV collagen. It has been confirmed that MMP-2 actively participates in cell invasion and metastasis. TGF-β2 is a growth factor with bidirectional regulatory effects on cell growth and differentiation according to different tissue sources, cell types, and conditions.^24^^,^^25^ In the physiological state, TGF-β2 is mainly latent in the aqueous humor in the form of inactive, which increases sharply under the stimulation of surgery and inflammatory response and the loss of lens homeostasis, eventually leading to later cataracts. PEDF is an angiogenic inhibitor, which can inhibit the formation of various new vessels. Studies have determined that PEDF is closely related to aging.^26^^,^^27^ In this study, PEDF levels in the aqueous humor in cataract patients were lower and MMP-2 and TGF-β2 levels were higher, indicating abnormal changes in PEDF, MMP-2, and TGF-β2 levels in the aqueous humor in cataract patients. The reason is that the body naturally produces more MMPs to degrade extracellular matrix accumulation, and MMP-2 abnormal expression in lens epithelial cells leads to the imbalance of synthesis and degradation of type IV collagen and laminin, the normal structure and function of the lens capsule are destroyed, leading to lens opacity.^28^ In addition, TGF-β2 is a polypeptide cell growth factor active in the anterior segment, and an abnormal increase in the level of aqueous humor can induce lens opacity.^29^

Pure senile cataract is the most common cause of reversible age-related blindness worldwide.^30^^,^^31^ Early diagnosis and appropriate therapeutic interventions are necessary to control the burden of this disease.^32^ One of the most commonly used subjective methods is the LOCS III.^33^ This method is based on retro-illumination slit-lamp images and has been regarded as valid since 1993.^34^ This well-known system is used in the diagnosis and treatment of cataract-related patients.^35^^,^^36^ Therefore, in this study, the authors used LOCS III to classify cataract disease severity, and the authors found that the level of PEDF was negatively correlated with LOCS III classification, and the levels of MMP-2 and TGF-β2 were positively correlated with LOCS III classification, suggesting that changes in the levels of the above indexes may be related to disease severity. It was further found that MMP-2 ≥ 15.13 pg/mL, TGF-β2 ≥ 385.91 pg/mL, and PEDF < 198.85 ng/mL were the risk factors for cataract development, suggesting that elevated levels of MMP-2 and TGF-β2 and lowered levels of PEDF may be involved in cataract development, mainly because PEDF is likely to act as a protective factor for lens epithelial cells, and the reduction of PEDF can aggravate the imbalance of stimulating factor/protective factor, further reduce the anti-stress and antioxidant ability of lens epithelial cells, increases the damage and apoptosis of lens epithelial cells, thus promoting lens aging and opacity.^37^^,^^38^ MMP-2 overexpression can cause the deposition of extracellular matrix on the posterior capsule of the lens and is therefore used as a scaffold for the continuous proliferation of the lens epithelial cells, leading to the destruction of the extracellular network structure and further affecting the function of the lens. TGF-β2 abnormally elevated level can effectively induce epithelial-mesenchymal transformation of human lymphatic endothelial cells and aggravate the opacity of the intraocular lens.^39^^,^^40^ In addition, the AUC value of the combined test to assess cataract development was greater than that of the PEDF, MMP-2, and TGF-β2 levels in the aqueous humor alone, suggesting that the combined test has an evaluation value for cataract development.

Overall, the changes in PEDF, MMP-2, and TGF-β2 levels in the aqueous humor of cataract patients are related to LOCS III classification, and the combined detection has an evaluation value for cataract development. However, there are limitations in this study. First, the sample size included in the study is small, and these conclusions need to be verified in a larger sample in the future. Also, the LOCS III method for classifying cataracts is an observer-dependent subjective tool that may cause measurement biases or inter-observer heterogeneity of results.

## Availability of data and materials

The datasets used and/or analyzed during the present study are available from the corresponding author upon reasonable request.

## Ethical approval

All procedures performed in this study involving human participants were in accordance with the ethical standards of the institutional and/or national research committee and with the 1964 Helsinki Declaration and its later amendments or comparable ethical standards. All subjects were approved by Longyan People's Hospital (n° 201910FJ202).

## Author's contributions

YongFeng Lin designed the research study. JinXia Xie and XiaoLuan Chen performed the research. YongFeng Lin provided help and advice. JinXia Xie and XiaoLuan Chen analyzed the data. YongFeng Lin wrote the manuscript. YongFeng Lin reviewed and edited the manuscript. All authors contributed to editorial changes in the manuscript. All authors read and approved the final manuscript.

## Funding

Not applicable.

## Declaration of competing interest

The authors declare no conflicts of interest.
